# Rapid diagnostic test supply chain and consumption study in Cabo Delgado, Mozambique: estimating stock shortages and identifying drivers of stock-outs

**DOI:** 10.1186/1475-2875-13-295

**Published:** 2014-08-02

**Authors:** Leah Hasselback, Jessica Crawford, Timoteo Chaluco, Sharanya Rajagopal, Wendy Prosser, Noel Watson

**Affiliations:** 1 Health Systems Group, VillageReach, Av. 25 de Setembro, 1123-11°Andar/E, Maputo, Mozambique; 2 Health Systems Group, VillageReach, PO Box 31348, Lilongwe 3, Malawi; 3 Health Systems Group, VillageReach, 2900 Eastlake Ave. E. Suite 230, Seattle, WA 98102, USA; 4 Department of Epidemiology, University of Washington, Seattle, WA 98195, USA; 5 OPS MEND, 43 Riverside Ave. Suite 103, Medford, MA 02155, USA

**Keywords:** Malaria, Stock-outs, Rapid diagnostic tests, Supply chain, Mozambique

## Abstract

**Background:**

Malaria rapid diagnostic tests (RDTs) are particularly useful in low-resource settings where follow-through on traditional laboratory diagnosis is challenging or lacking. The availability of these tests depends on supply chain processes within the distribution system. In Mozambique, stock-outs of malaria RDTs are fairly common at health facilities. A longitudinal cross-sectional study was conducted to evaluate drivers of stock shortages in the Cabo Delgado province.

**Methods:**

Data were collected from purposively sampled health facilities, using monthly cross-sectional surveys between October 2011 and May 2012. Estimates of lost consumption (consumption not met due to stock-outs) served as the primary quantitative indicator of stock shortages. This is a better measure of the magnitude of stock-outs than binary indicators that only measure frequency of stock-outs at a given facility. Using a case study based methodology, distribution system characteristics were qualitatively analysed to examine causes of stock-outs at the provincial, district and health centre levels.

**Results:**

15 health facilities were surveyed over 120 time points. Stock-out patterns varied by data source; average monthly proportions of 59%, 17% and 17% of health centres reported a stock-out on stock cards, laboratory and pharmacy forms, respectively. Estimates of lost consumption percentage were significantly high; ranging from 0% to 149%; with a weighted average of 78%. Each ten-unit increase in monthly-observed consumption was associated with a nine-unit increase in lost consumption percentage indicating that higher rates of stock-outs occurred at higher levels of observed consumption. Causes of stock-outs included inaccurate tracking of lost consumption, insufficient sophistication in inventory management and replenishment, and poor process compliance by facility workers, all arguably stemming from inadequate attention to the design and implementation of the distribution system.

**Conclusions:**

Substantially high levels of RDT stock-outs were found in Cabo Delgado. Study findings point to a supply chain with a commendable degree of sophistication. However, insufficient attention paid to system design and implementation resulted in deteriorating performance in areas of increased need. In such settings fast moving commodities like malaria RDTs can call attention to supply chain vulnerabilities, the findings from which can be used to address other slower moving health commodities.

## Background

The timely diagnosis and appropriate treatment of malaria constitute essential components of effective malaria case-management. With regard to diagnosis, malaria rapid diagnostic tests (RDTs) are particularly useful in low-resource settings where follow-through on traditional laboratory diagnosis is challenging or lacking. RDTs simplify the processes and capacity needed to extend services to populations in remote, underserved areas. At peripheral health facilities, RDT-based diagnosis of malaria is strongly recommended by the World Health Organization (WHO) as it does not require laboratory infrastructure or highly skilled professionals unlike the microscopy-based diagnosis [1].

**Figure 1:**
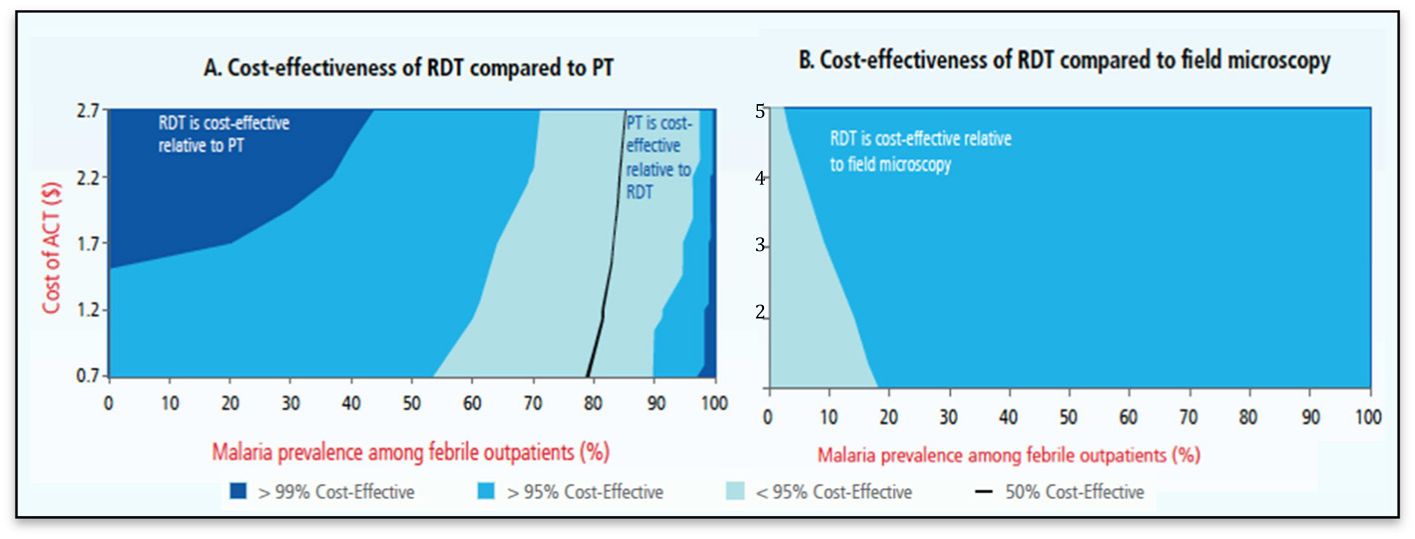
**Cost-effectiveness of RDTs compared to (a) presumptive treatment and (b) field microscopy**[1]**.**

The cost-effectiveness of RDTs has been extensively evaluated in various settings in sub-Saharan Africa [2-5]. Figure 1 illustrates the cost-effectiveness of malaria RDTs (at a cost of USD 0.60 per test) using the WHO-decision tree analysis across a wide range of malaria prevalence estimates [1]. At a cost per ACT (Artemisinin-based combination therapy) adult dose of USD 1.70, RDTs are considered cost-effective below a threshold of 64% malaria prevalence [1]. These figures imply that in the majority of African settings RDTs would be cost-effective compared to field microscopy or symptom-based presumptive treatment (PT). In Mozambique, RDTs are commonly used to diagnose malaria. In a study of RDT cost-effectiveness in southern Mozambique, Zikusooka *et al.* reported total cost savings between USD 1,485 and USD 16,908 when using RDTs for definitive malaria diagnosis in populations where 25% of febrile cases were RDT positive [6].

The continuous and universal availability of anti-malarials and RDTs is central to addressing the global burden of malaria. Prior research suggests high levels of stock-outs of both types of commodities [7-9]. There is limited research examining the drivers of such stock-outs in Africa. Sudoi *et al.* have recently reported on the persistence of high levels of artemether-lumefantrine (AL) stock-outs at health facilities in Kenya [10]. Similarly, Blanas *et al.* while not formally assessing the causes of ACT and RDT stock-outs for community health workers in Senegal, attribute these to inadequate supplies at the pharmacy and district health centres [11]. Neither study exclusively examines the drivers of RDT stock-outs. As with other health commodities, the availability of RDTs for use at a health centre depends on the distribution system and processes within the health system. However as diagnostics, RDTs are expected to be used at greater volumes than other health commodities routinely used for treatment. RDTs therefore have characteristics of so called fast moving products and thereby pose greater challenges for being kept in stock in a supply chain [12].

In 2008, a number of challenges in the RDT supply chain in Mozambique were identified that impacted test availability [13]. Firstly, coordination amongst various stakeholders in the health system – pharmacy, laboratory, and malaria programs as well as the provincial ministry of health (DPS: Direcção Provincial da Saúde) management - was found to be poor and inconsistent. Secondly, there was little-to-no consumption information from the health centres making its way to higher levels of the supply chain. Thirdly, the reporting system at health facilities was found to be fraught with data quality issues in the absence of a nationally standardized form for data collection.

Despite the above challenges identified in the supply chain in Mozambique, there is still limited information on the extent and additional causes of stock-outs in Mozambique. Therefore, a cross-sectional study was undertaken to estimate malaria RDT stock shortages and the percentage of overall need met by the existing stock in the Cabo Delgado province of Mozambique. Additionally, drivers of stock-outs of RDTs for malaria were assessed and underlying distribution system characteristics examined.

## Methods

The approach for this research involved a combination of quantitative analytical methods and case study research methods. Both categories of methods are described.

### Quantitative methodology

#### Sampling

**Figure 2:**
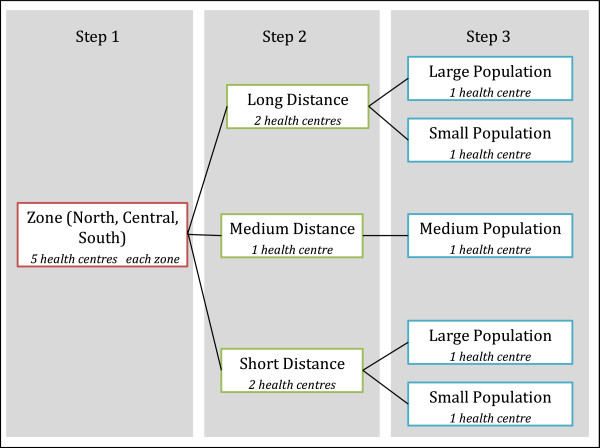
Purposive sampling methodology.

A purposive sampling technique was used to select fifteen health centres (15% of facilities) in the Cabo Delgado province. The sample size was selected based on feasibility and budgetary considerations. However, the purposive sampling strategy permitted the selection of a representative sample of health centres. The health centres were selected to represent a variety of distances from the provincial store and population catchment sizes as shown in Figure 2. The distance to health centres was categorized according to their accessibility and the corresponding commute time taken to reach each health centre. The estimated catchment population sizes were divided into 3 groups. A size of 20,000 or below was considered a small population, medium ranged from 20,000 through 25,000 and large populations were 25,000 or more residents in the catchment areas. The sampling frame consisted of all facilities in the province with a history of reliable data collection using routine forms. The facilities were stratified according to a three-step process as shown in Figure 2. Firstly, they were categorized into three zones with five facilities in each; which were additionally stratified based on their proximity to the provincial store. In the third and final step, the facilities were further sub-divided according to their catchment sizes.

#### Quantitative indicators

Stock shortages at each health facility were estimated using the measure of ‘lost consumption’. A lost consumption event reflects the unavailability of the malaria RDT at a health centre due to a stock-out. Lost consumption represents the percentage of overall need for RDTs that was not met by the health facilities with their available stock of RDTs. A range of lost consumption estimates are reported, followed by an average estimate weighted by available months of consumption data from each health centre.

To generate lost consumption measures it was crucial to generate estimates of the true RDT consumption that would have occurred if inventory was available. Research from the for-profit sector shows that using consumption data from products faced with stock-outs is not a good measure of its true demand rate [14,15]. The same can be said for public health supply chains; not knowing the true demand rate complicates forecasting and stock delivery. The true or expected RDT consumption at a health centre was estimated based on the consumption during days/weeks of full stock. This was applied to the number of days a health centre could have been out of stock to estimate the true consumption for the entire study period. Due to data limitations, it was not always possible to identify the days in which the health centre was definitely out of stock, which would have been preferred. Instead the days that a health centre could have been out of stock were identified by time periods that ended with no inventory available implying that inventory ran out at some point during the time period. The estimate of true RDT consumption at each health centre over the period of the study was then compared to the observed consumption to determine the percent of lost consumption due to stock-outs (see Additional file 1 for more details).

#### Data collection and analysis

Consumption and stock-out data were collected from each health centre on a monthly basis from October 2011 – May 2012. Primary data sources included routine forms and reports used in health facilities such as pharmacy forms, laboratory forms and stock cards. Pharmacy forms were introduced at health centres in August 2011 and completed on a monthly basis to report information on stock levels at the beginning and end of each month, and both receipts and consumption during the month. In addition to the above information, laboratory forms reported monthly data on test results. Stock cards were maintained in the health centre storeroom and were updated throughout the month with information on the quantity of RDTs received and issued, stock on hand, date of transaction, and the location of issue (triage or maternity). Data collected from all fifteen health centres were used in the quantitative analysis.

Stock shortages were quantitatively evaluated by estimating lost consumption at each health facility. This was done using a forecasting methodology wherein the true consumption rate was estimated during periods of no stock-outs at a given health facility using one of three approaches, based on data availability (see Additional file 1). Stock cards were used as the primary data source for stock levels throughout the month due to the frequency at which they are updated. Accurate data from stock cards was not always available at each of the facilities and in those cases, data from lab and pharmacy forms were the only alternative. Estimation of true consumption was further complicated since malaria RDTs were used both at triage and the maternity ward at all facilities. Since issues from the storeroom were made in units of 25 RDTs each, there could be an event where the storeroom and triage were out of stock but the maternity ward had RDTs for supply. Each department would conceivably have its own true consumption rate, which could shift to the other area in the event of a local stock-out. The departmental true consumption was extrapolated in order to estimate the expected consumption rate for the facility as a whole during monitoring periods which could have had stock-outs identified by a zero inventory balance at the end of the period. Lost consumption was estimated as a percentage by comparing the true or expected consumption for the period of the study to the observed consumption rate at each facility (see Additional file 1).

Generally this approach to forecasting would tend to result in an underestimate of the true consumption since periods of stock-outs would have higher levels of demand than periods of non stock-outs *ceteris paribus,* and even more so for products exhibiting seasonality like malaria commodities. In particular the rainy season in Mozambique spans October to March, which overlapped with the time period of data collection.

Data were analysed using Excel and R. The relationship between the lost consumption rate and the rate of monthly consumption was analysed using linear regression. Since there was considerable variation in the number of months of data available from each facility, linear regression models and Pearson’s correlation coefficient were also used to assess the effect of this parameter on the lost consumption estimates.

#### Case study research methodology

In addition to identifying the extent of lost consumption accompanying stock-outs, the primary purpose of this research was to identify factors such as distribution system characteristics that could affect RDT stock availability at the health centres. A case study based approach was employed to understand the mechanisms governing the management of the entire health supply chain for malaria RDTs and their effect on stock availability.

During the study period, physical storage of RDTs within the Cabo Delgado province took place at the provincial and district stores and at heath centres. Management of the health supply chain for RDTs not only involved personnel at these storage locations but also both i) general health supply chain personnel responsible for designing and administration of the health supply chain and ii) malaria control programme support personnel with specific responsibilities for malaria commodities such as procurement. Over a one-week period, interviews were conducted with key informants at: health supply chain technical support, malaria control program support, Mozambique’s central medical store (Central De Medicamentos e Artigos Médicos - CMAM), provincial medicines warehouse district medical store and district medical directorship. Four health centres in the larger sample - Namanhumir, Catapua, Nakoto and Mushara - were visited and staff members were interviewed. Qualitative data were collected through semi-structured interviews on RDT use, supply chain processes, data collection, and stock management. Supported by a framework of themes described below rather than an explicit questionnaire, interviewers were allowed to pursue issues as raised by the interviewee. Stock and clinical records related to RDTs were also reviewed at each site. These records included the data analysed in the quantitative section plus additional records available at the site.

The qualitative and quantitative data collected from the case study were analysed with a focus on three areas - supply system, data quality and availability, and leadership in supply chain management – and their subcomponents as captured in Table 1. These three areas and their components were considered broad enough to comprehensively guide the search for drivers of stock-outs and served as the foundation for the themes used to support the semi-structured interviews. In analysing the data collected from the case study, the qualitative description was initially coded using these areas and subcomponents. This coding was refined and revised based on discussion between the researchers. In particular it became apparent that a distinction was needed between the intended design of the system and the reality of its execution where they differed. Identification of factors that would drive stock-outs was then primarily based on empirical observations of deviations from intended supply chain management design but also on the quality of the intended design in the first place. The quality of the intended design was evaluated by considering whether the components of the supply chain design were a logical fit to each other and the context, and also taking into account best practices from other healthcare and non-health care supply chain settings and research.

**Table 1:** Outline of data analysis

**Areas of analysis**	**Focus**
Data quality and availability	■ Relevant types of data
	■ Cost/effort to obtain
	■ Accuracy (hidden defects)
	■ Motivation to share
	■ Data appropriate for decision making
Decision makers	■ Identification
	■ Competence
	■ Alignment of motivation
	■ Synchronization of actions
	■ Behavioural factors
Supply system	■ System Areas (Warehousing, Transportation, Distribution, Forecasting, Coordination/Planning)
	■ Performance Drivers (capability, infrastructure, control mechanisms)
	■ Complexity
	■ Degree of Structure (expectations for stable patterns and relationships)
	■ Moving parts (number of actors, events or activities that contribute to consumption of health commodity)

## Results

In total, 15 facilities were surveyed over 120 data collection units (a data collection unit represents one month of data from one health centre). Stock-out patterns for the malaria RDT varied according to data sources as shown in Table 2. According to the laboratory and pharmacy forms, an average proportion of 17% of health centres experienced a stock-out on any given month across the 8-month period. The stock cards showed a much higher proportion of health centres stock-out averaging 59%. Likewise, monthly stock-out patterns captured by the three data sources indicated that stock cards reflected a much higher percentage of health centres reporting stock-outs for most months. Stock cards are likely the most reliable data source because they are completed throughout the month whereas the laboratory and pharmacy forms are completed on specific dates indicating levels of existing stock on that date.

**Table 2:** Proportion of health centres reporting stock-outs of malaria RDTs

**Months**	**Data sources**		
	**Laboratory form (95% CI)**	**Pharmacy form (95% CI)**	**Stock card (95% CI)**
Overall	17%	17%	59%
October	33% (8, 58)	9% (0, 25)	27% (2, 52)
November	15% (0, 33)	10% (0, 28)	45% (17, 73)
December	46% (20, 72)	13% (0, 36)	64% (37, 91)
January	43% (19, 67)	33% (3, 63)	91% (75, 107)
February	0%	0%	64% (37, 91)
March	0%	25% (0, 54)	55% (27, 83)
April	0%	22% (0, 48)	73% (48, 98)
May	0%	22% (0, 48)	55% (27, 83)

### Estimation of lost consumption

**Figure 3:**
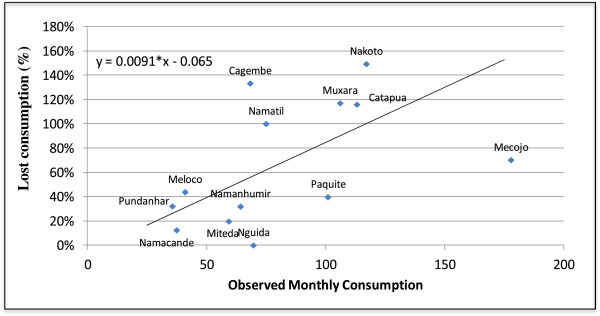
Lost consumption versus observed monthly consumption of malaria RDTs.

The study found significantly high levels of lost consumption percentage estimates for RDTs for malaria ranging from 0 to 149% with an average of 78%, weighted by consumption (Table 3). The approached used to forecast lost consumption does not lend itself to a confidence interval around the estimate, however as discussed earlier it is likely to underestimate the lost consumption percentage. Lost consumption percentage estimates for 13 health centres are given in Table 3. Two of the health centres from the study had insufficient data for estimation of lost consumption. The lost consumption percentage was plotted versus observed monthly consumption (Figure 3.). At the 5% significance level, regression analysis indicated a significant positive association between lost consumption percentage and observed monthly consumption; for a 10-unit increase in observed monthly consumption, lost consumption percentage was found to increase by 9 units (95% CI: 1.7, 16.5; Figure 3). This is an important finding; in facilities with higher levels of true consumption, more of the true demand is not being met, either in magnitude or in proportion. If size of consumption reflects critical malaria need, the findings indicate that the supply processes for RDTs are increasingly deficient for areas of critical need.

**Table 3:** Lost consumption percentages of malaria RDTs by health centre

**Health centre**	**Approach**	**Observed monthly consumption**	**Number of months available for analysis**	**Lost consumption**
Catapua	1	113	7.3	116%
Namanhumir	1	64	7.0	32%
Mecojo	1	178	8.0	70%
Nakoto	1	117	6.3	149%
Paquite	1	101	8.1	40%
Meloco	2	41	3.3	44%
Nguida	2	70	5.2	0%
Namacande	2	37	7.1	12%
Muxara	1	106	6.1	117%
Pundanhar	2	36	8.1	32%
Miteda	2	59	8.1	20%
Cagembe	3	68	7	133%
Namatil	3	75	6	100%

Despite the variation in the number of months of data available from each health centre, there was little evidence to suggest that this had a systematic effect on lost consumption forecasts. The Pearson’s correlation coefficient between the variables was not found to be significantly different from zero. Additionally, linear regression models that included number of months of data available as explanatory variables for lost consumption percentage were rejected in favor of models without it.

### Drivers of shortages

The qualitative analysis identified drivers of shortages for RDTs within all three areas evaluated – supply system, data quality and accuracy, and leadership in supply chain management. Firstly, the health supply system for RDTs in the national context of donor support, and its use of consumption data is described below.

Since their introduction in Mozambique in 2007, RDTs have been generally well received by health professionals because of the faster results and ensuing quicker transition to treatment options. In August 2011, the supply management system for RDTs changed from a traditional requisition-based supply to a consumption driven resupply. In the latter, replenishment quantities to health centres and districts were determined by the facility one level higher in the supply chain - the district store and provincial store respectively. Integral to this approach was the sharing of consumption data from health centre with provincial and district staff. Specifically, health centres sent their monthly consumption and inventory data to the district and the district sent their information to the province. In addition, explicit formulas based on monthly consumption were introduced to aid calculation of quantities to be delivered to a particular health centre by a district, or to a district by the province. Health centre and district monthly replenishment quantity was based on an order up-to mechanism using the following calculation: 2 × monthly consumption – existing stock; where 2 × monthly consumption was the target up to which inventory was replenished. In addition, a similar formula guided the provincial store in making requisitions to CMAM. From the province to CMAM, stock was replenished quarterly so the order up-to target was six months of stock instead of two. Finally when a stock-out occurred at a health centre, staff either placed an emergency requisition to the district or wait until the next monthly distribution. The latter often occurred when the health worker suspected or knew that the district did not have stock to provide.

According to interviews with key stakeholders, malaria RDTs in Mozambique are funded by a combination of donors including the World Bank, Global Fund and United States Government. Forecasting for malaria tests was done by a USAID|DELIVER team using a morbidity approach with adjustments for access to medical care and treatment-seeking behaviour. After the initial forecast, a supply procurement plan for a 12-month period was created to provide the timing of procured quantities. This reflected factors such as seasonality and timing of specific malaria related programmes and policy changes. Procurement lead-times for malaria tests were approximately four months and the USAID | DELIVER procurement team shared revised procurement plans with donors to drive alignment of fund flows, an accomplishment that was challenging.

The existing RDT health supply chain in Cabo Delgado has several strengths that place the drivers of shortages in context. First, the health commodity is well accepted by facility workers. Second, procurement for tests was donor supported, managed by third parties with significant experience in procurement for developing countries and sensitive to aggregate supply needs as reported by CMAM. Third, a requisition-based supply chain has been associated with supply dysfunction [16,17]. Supply chains need to respond to timely consumption data to ensure that inventory are appropriately stocked with respect to demand [18,19]. In some cases, the consumption data aids the supply chain in forecasting future demand. Requisitions can be distorted signals of consumption especially at higher levels of the supply chain. This results in cycles of stock-out followed by periods of excessive inventory. A reduced reliance on requisitions mitigates this distortion since resupply decisions are shifted upstream in the supply chain. In the case of health centres, this circumvents the need for health workers to be competent in inventory resupply quantification skills and relieves them to focus on delivering care. Additionally, it might be more optimal to allocate and develop such skills at the district level or upstream at the supply chain, thereby benefiting several health centres downstream. The system introduced in Cabo Delgado deserves commendation for its sophistication, especially as it addresses the issues that had been identified as affecting test availability – sharing of consumption data, standardized forms and coordination [13]. Despite these strengths, several features were identified in the health supply chain in Cabo Delgado that contributed to frequent stock-outs of malaria RDTs.

The first driver of stock-outs was linked to data quality and availability. Poor tracking of lost consumption lowered the accuracy of consumption data provided to the consumption-based resupply system. In Cabo Delgado, consumption reports from health centres only captured actual consumption. The Chief Pharmacist at the Provincial Stores pointed out that consumption reports should include an annotation about the number of days over which consumption occurred so that lost consumption could be imputed. However, none of the forms requested this information and data on lost consumption was rarely shared. This was additionally compromised by the fact that the district shared an aggregated summary of all health centre consumption data with the province. Due to the lack of tracking lost consumption, the system responded to an underestimate of the true demand thereby positioning lower inventory than needed in the supply chain.

Three drivers were identified within the supply system design and execution. First, there was a mismatch between the sophistication of control mechanisms for inventory replenishment and the reality of supply chain execution. In Cabo Delgado, the original expectation for resupply lead-times was within two weeks. However actual lead-times were greater than two weeks. Typically, resupply would not occur until sufficient inventory arrived at the higher level. This lengthening of lead-times implied that inventory levels across the entire supply chain were generally lower than they should be. However, the formulas used for inventory replenishment were simple order-up-to mechanisms, which sought to bring inventory levels up to a multiple of the monthly consumption. These formulas only focused on local inventory; the inventory at a single tier of the supply chain. Thus with no control mechanisms that considered the global inventory levels, these local (and thus myopic) mechanisms were misaligned across the supply chain without any opportunities for realignment [17,18]. For example, bringing district inventory levels up to their local target may be insufficient if the health centres below the district are under-stocked frequently. Cabo Delgado lacked the additional sophistication in inventory control mechanisms that could address this potential misalignment.

Second, there were inadequate or poor processes in place for addressing seasonality in consumption of RDTs due to the effect of the rainy season on malaria prevalence. The timing of Mozambique’s rainy season and associated transportation difficulties as well as its general effects on volume of malaria patient arrivals at health centres are reasonably well known among Cabo Delgado’s health supply system administrators. Facility workers estimated that the rainy season could increase RDT use by as much as 300% for some facilities. However, apart from the procurement agents incorporating seasonality in their procurement plans, there was no other additional preparation for seasonality such as a ramp-up in inventory of tests before the rainy season.

The order up-to mechanisms used for the replenishment process had limited considerations for seasonal changes. Since it is based on consumption, as the consumption increases, so does the replenishment quantity. However, because it is a monthly process, there is one month’s delay in the effect of increased consumption in replenishment amounts. In addition, when these increased replenishment amounts are needed, inventory would need to have been positioned in advanced at the district or province in order to meet these needs. Such advanced positioning of inventory is not compatible with the simple order up-to mechanism used, as these mechanisms did not look forward in anticipation of the significant increase in months of consumption that would result from seasonal changes. Additionally, if a health centre stocks out of RDTs at the start of the seasonal increase, the consumption is limited to consumption up to the point of stock-out, which then limits the replenishment amount.

Seasonality is one of the factors that reduces the appropriateness of a consumption driven supply system and even more so, a system like Cabo Delgado’s that suffers from effects of longer than expected lead times and poor tracking of lost consumption.

Third, poor process compliance in information collection and sharing, and inventory management was observed in facility workers, as demonstrated by the differing data collected by the three data sources. System processes that involving health workers at health centres such as generating consumption reports, keeping patient registers and tick-charts, timely emergency requisitions, and expired health commodity tracking and disposal, require their attention and competence to enable proper functioning of the supply chain. In Cabo Delgado however, a mass training for health workers in supply system processes had taken place in 2005 and an ongoing supervision of worker process compliance was lacking.

Finally, these drivers can be considered symptoms of a larger issue: poor leadership in system design and implementation. The drivers of shortages discussed above suggest that attention to system design and implementation could be improved. Additional examples of system inefficiencies which support this conclusion include: i) multiple consumption forms found in various health centres suggest successive revisions of the original form that was poorly tested before implementation, ii) no official form for tracking consumption of malaria tests, instead facility workers created their own tick sheets, and iii) an emergency requisition process that was a carryover from the previous requisition based resupply system, and required a different set of steps with which facility workers were not familiar, given the new consumption based resupply system.

## Discussion

This is the first study of its kind to evaluate stock shortages of malaria RDTs in a developing country setting. This study reveals important findings on drivers of lost consumption and stock shortages. Health facilities reported significantly high levels of lost consumption due to stock-outs, with an average of 78%. In addition, higher levels of the lost consumption rate were found to be associated with facilities that observed higher monthly consumption. On average, the health centres in this study stocked out of RDTs roughly 50% of the time during the eight-month study period.

Study findings point to a supply system with significant gaps and challenges, indicating a system design that was not entirely appropriate in the context of Cabo Delgado. To date, few studies have examined supply chain characteristics and factors that affect stock levels and availability of essential health commodities at health centres in sub-Saharan Africa. However several studies have reported widespread stock-outs of anti-malarials in Africa [10,20,21]. Drivers of ACT stock-outs were similar to those identified in the present study: suboptimal supply, inaccurate inventory of existing stock in stock cards, and poor system design.

The results of this study serve as recommendations for the design of the supply system so as to improve i) the quality of execution within the supply system, including making data available for decision-making, and ii) the quality of decision making within the system. Crucial for this design however, is the ability of the system to both recognize when performance deviates from expectations and implement steps towards rectifying this deviation. This is particularly important for fast moving products, as reducing the time that the system operates outside of expectations should reduce the effects of poor supply. But even for slower moving items, particularly in resource-constrained settings, this would help minimize the wastage of scarce resources.

Examples of approaches that address data availability and execution within the supply system include Burkina Faso where the Ministry of Health adopted a transversal approach to improving health logistics through the deployment of logistic professionals [22]. The primary responsibilities of the logistic professionals involve strengthening communication and coordination across various levels of the supply chain, organizing logistics for health emergencies, and assisting other health professionals like pharmacists and doctors in improving supply chain logistics. Task shifting approaches such as these, as recommended by the WHO, can alleviate the administrative burden on frontline staff while increasing the access to, and availability of health commodities.

Approaches to improving the quality of decision-making within the supply system include increased sophistication in the policies for managing resources like inventory. Such sophistication should address issues such as seasonality and inventory maintenance across multiple levels. In an exploratory study of factors affecting the performance and use of malaria RDTs in South Africa, Moonasar *et al.* found sporadic stock-outs of RDTs at 20 health facilities during peak transmission seasons [20]. In contrast, this was a critical concern for the Cabo Delgado supply chain where existing processes did not adequately ramp-up stock levels in preparation for the rainy season. The high volumes of demand and potential transportation disruptions are more appropriately addressed by strategies such as staging buffer inventory at health centres in anticipation of these events. In addition, improved algorithms for forecasting and inventory replenishment should take seasonality into account.

The ability to recognize when system performance is outside of expectations and to respond appropriately is both an attitude and an organization of system components. In particular, it is an attitude, because it is usually easier to accept deviations from expectations rather than maintain sensitivity to them. However such an attitude has been argued in operations management literature as crucial to maintaining system performance and over time improving performance through learning [23]. Such attitudes are generally driven by senior level management. This implies that in resource-constrained settings, stock-outs will persist if their presence is explicitly or tacitly accepted and if this acceptance is portrayed by senior administrators of the health system.

In addition to a change in attitude towards stock-outs, deviations from system functioning need to be measured and communicated to decision makers in a timely manner so that a response can be initiated. For example in Tanzania, the ILS gateway was established; a system based on short message service (SMS) that generates consistent and frequent inventory and consumption data on a small group of essential commodities from health facilities across the country [24]. It has been credited with improving the availability of these commodities. However data availability alone, without addressing issues raised by the data cannot improve the availability of the commodities.

Finally, an attitude of responding to deviations from system expectations, implies that system design may be an ongoing process. Such a perspective runs counter to more static system design approaches that seek to cement system attributes during implementation. Cabo Delgado showed some signs of ongoing system redesign, for example, the different designs of forms used at various facilities. However such redesign efforts are compromised when their results are not uniformly applied across the supply chain.

The recently approved Mozambique Ministry of Health Strategic Plan for Pharmaceutical Logistics shares features of our recommendations. The plan correctly notes that system optimization is a long process that must adapt to the current realities both at systemic and country-level. The plan incorporates several concepts of system optimization, including streamlined warehouse storage that will shorten the response cycle to guarantee prompt deliveries, minimize stock-outs and distribution costs. The plan also calls for improved collection and analysis of consumption data.

### Limitations

A number of challenges to data quality and collection at the health centre level impacted overall data availability and accuracy. Data from primary sources like laboratory forms, pharmacy forms and stock cards were not consistently available at health centres. In addition there was large variation in the types of forms used to collect RDT consumption information and the ways in which the forms were used at health centres. The study team also noted a high frequency of mathematical errors in the primary data sources. Additionally, the study was implemented during the rainy season, which is the peak season for malaria; true consumption is expected to be lower in the off-season.

## Conclusions

Fast-moving commodities such as RDTs tend to stress and reveal vulnerabilities in supply chains. This has implications for system design and the leadership required to improve efficiencies in distribution, data collection and utilization eventually leading to a reduction in stock-outs. In a broader sense, the lessons learned from the study of these commodities can be extrapolated to other health commodities that, although slower moving, may not be as adequately funded. The latter require greater optimization of supply chain processes to avoid wastage and ensure efficient resource allocation. Lastly, the study findings are broadly applicable to the distribution system in Cabo Delgado due to the purposive sampling of health centres from this region.

## Abbreviations

ACT: Artemisinin-based combination therapy; AL: Artemether-lumefantrine; CMAM: Central De Medicamentos e Artigos Médicos; DPM: Deposito Provincial de Medicamentos; DPS: Direcção Provincial da Saúde; PT: Presumptive treatment; RDT: Rapid diagnostic test; WHO: World Health Organization.

## Competing interests

The authors declare that they have no competing interests; financial or otherwise.

## Authors’ contributions

NW and LH conceived of and designed the study. TC designed the data collection tools. NW, LH, JC and SR conducted data analysis and interpreted the findings. NW, SR and WP drafted and finalized the manuscript. All authors have read and approved the final manuscript.

## Supplementary Material

Additional file 1 A detailed description of the methodology used to estimate the monthly true consumption rate at each health facility. Click here for file
