# Supplementary material for: Rapid diagnostic test supply chain and consumption study in Cabo Delgado, Mozambique: estimating stock shortages and identifying drivers of stock-outs
Source: Malar J. 2014 Aug 2;13:295. doi: 10.1186/1475-2875-13-295 (PMC4237853; doi:10.1186/1475-2875-13-295)
Supplement: Additional file 1 — A detailed description of the methodology used to estimate the monthly true consumption rate at each health facility. [file 1475-2875-13-295-S1.docx]

## Additional File 1: Methodology for the Estimation of lost and true consumption

The forecasting methodology for lost consumption is based on a very basic approach of estimating a true consumption rate during periods of demand, e.g., days, for which we are fairly certain that there were no stock-outs. The true consumption rate (TCR) was then extrapolated to an expected consumption during periods of stock-outs. This estimated consumption was then compared to the observed consumption to determine lost consumption due to stock-outs.

Let NOSTKOUT and STKOUT be the period in days with no stock-outs and with stock-outs, respectively at a health center. (In practice, these parameters were typically the number of days respectively where *we were sure* there were no stock-outs and *there could have been* some stock-outs.) Also let STKOUTCONS and NOSTKOUTCONS be the observed consumption in units of commodities, respectively during periods of no stock-outs and those with stock-outs at the health center. Our forecasting approach estimates in units of commodities, i) the TCR at the health center, and ii) an estimated consumption for periods with stock-outs, ESTCONSTK. The estimated consumption at the health center for the entire period, ESTCONS, and the observed consumption at the health center for the entire period, OBSCONS, can then be given by the following:

$$ESTCONS=ESTCONSTK+NOSTKOUTCONS,$$

$$OBSCONS=STKOUTCONS+NOSTKOUTCONS.$$

Finally a lost consumption percentage LSTCONS% is determined by the following:

$$LSTCONS\%=\frac{ESTCONS}{OBSCONS}.*100$$

### Estimating True Consumption

The approach used to estimate the TCR varied with data availability. Three distinct approaches were used.

#### Approach 1

This approach was used when stock cards showed multiple flows from the stock room to more than one department, e.g., SMI and Triage, over the period of demand. Since stock-out and consumption are specific to each department it is crucial to track them separately when possible. More commonly, data at health centers was sufficient to track consumption at two departments only; namely Triage and SMI. Flows from stockroom to a department, d=Triage or SMI, were used as a proxy for consumption at the department from which NOSTKOUTCONS_d_ and NOSTKOUT_d_ were calculated. The consumption rate TCR_d_ at each department was calculated as follows:

$${TCR}_{d}=\frac{{NOSTKOUTCONS}_{d}}{{NOSTKOUT}_{d}}.$$

#### Approach 2

This approach was used when the stock cards showed inventory flows primarily to one department but sparse flows to any other departments. In this case it was not feasible to estimate consumption flows for other departments. Consequently, the forecasting methodology ignored departments with sparse records and instead assumed all inventory to have flown to the primary department. The TCR for the primary department was then calculated using the formula for TCR_d_ in Approach 1.

#### Approaches 3a & 3b

Two similar approaches were used when the stock cards were sparse or nonexistent for any departments within the health center but data on consumption was available from lab/pharmacy forms. Using data from months where commodities were available both at the beginning and end of the month, the TCR was calculated from the formula for TCR_d_ in Approach 1. We refer to this approach as Approach 3a.

In cases where all months showed stock-outs, the TCR cannot be calculated theoretically. Hence months with consumption or with high levels of consumption comparatively were considered as substitute for periods without stock-outs (Approach 3b). The TCR was then calculated using these months. Even though this approach is inaccurate, it is expected to consistently underestimate the TCR and lost consumption.

### Estimating lost consumption

The approach for estimating lost consumption was basically similar to those described above. The TCR was extrapolated over the period during which there were stock-outs, as follows:

$$ESTCONSTK=TCR\times STKOUT.$$

One major change to this basic approach was when stock room records showed multiple flows from the stock room to more than one department, e.g., SMI and Triage. This case corresponds to Approach 1 in estimating true consumption. Interviews with health center staff revealed that departments were sufficiently close to each other; so when one department stocked out of a diagnostic test patients would be sent to another department. This substitution implies that the true consumption rate of either department TCR_d_ also included some consumption that had originally arrived at the other department; consumption that we would have considered lost if we did not account for substitution. Thus ignoring substitution would have resulted in an over-estimate of the lost consumption at a health center. To adjust for this substitution we used a heuristics based on a proxy for the probabilities that each health center was stocked out and an assumption that these probabilities were independent. (It is unlikely that the probabilities are independent because the departments have the same inventory source for supply, but our approach would provide a conservative estimate)

Specifically, when one department such as Triage, is stocked out, and the other is not, then the true consumption during that period at SMI and the observed consumption at Triage capture the full consumption during that period. Let v_d_ be the probability that health center department d=(T)riage or (S)MI, has stocked out. The above observation leads to the following formulation for estimated consumption for the health center, ESTCONS, based on the individual estimated consumption ESTCONS_d_ and observed consumption OBSCONS_d_ for each department:

$$ESTCONS=v_{T}\left( 1-v_{S} \right)\left( {ESTCONS}_{S}+{OBSCONS}_{T} \right)+v_{S}\left( 1-v_{T} \right)\left( {ESTCONS}_{T}+{OBSCONS}_{S} \right)+v_{T}v_{S}\left( {ESTCONS}_{S}+{ESTCONS}_{T} \right)+\left( 1-v_{T} \right)\left( 1-v_{S} \right)\left( {OBSCONS}_{S}+{OBSCONS}_{T} \right)$$

Note that the formulation is based indirectly on true consumption rates (through ESTCONS) instead of TCR directly since the total time periods used for each department in our data sample were not always identical. Finally as a proxy for the probability that a health center had stocked out we used the percentage of days that were stocked out at each department for probability v_d_

$$v_{d}=\frac{{STKOUT}_{d}}{{STKOUT}_{d}+{NOSTKOUT}_{d}}$$
